# Development of dietary assessment instruments which can take cultural diversity and dietary acculturation into account: eating in Sweden (‘Mat i Sverige’)

**DOI:** 10.1017/jns.2024.72

**Published:** 2024-11-15

**Authors:** Marleen A. H. Lentjes, Sarah Lönnström, Karin Lobenius Palmér, Zeinab Alsammarraie, Anna Karin Lindroos, Jessica Petrelius Sipinen, Afsaneh Koochek, Robert Jan Brummer, Scott Montgomery

**Affiliations:** 1 School of Medical Sciences, Faculty of Medicine and Health, Örebro University, Örebro, Sweden; 2 Clinical Epidemiology and Biostatistics, School of Medical Sciences, Faculty of Medicine and Health, Örebro University, Örebro, Sweden; 3 Division for Risk and Benefit Assessment, Swedish Food Agency, Uppsala, Sweden; 4 Department of Internal Medicine and Clinical Nutrition, the Sahlgrenska Academy, University of Gothenburg, Gothenburg, Sweden; 5 Department of Food Studies, Nutrition and Dietetics, Uppsala University, Uppsala, Sweden; 6 Clinical Epidemiology Division, Karolinska Institute, Stockholm, Sweden; 7 Department of Epidemiology and Public Health, University College London, London, UK

**Keywords:** 24h-diet recall, Content validity, Culture-specific food, Dietary acculturation, Dietary assessment

## Abstract

Since lack of culture-specific foods in dietary assessment methods may bias reported dietary intake, we identified foods and dishes consumed by residents not born in Sweden and describe consequences for reported foods and nutrient intake using a culturally adapted dietary assessment method. Design consisted of cross-sectional data collection using (semi-)qualitative methods of dietary assessment (and national diet survey instrument *RiksmatenFlex*) with subsequent longitudinal data collection using quantitative methods for method comparison (December 2020–January 2023). Three community-based research groups were recruited that consisted of mothers born in Sweden, Syria/Iraq, and Somalia, with a median age of 34, 37, and 36 years, respectively. Women born in Syria/Iraq and Somalia who had lived in Sweden for approximately 10 years, reported 78 foods to be added to *RiksmatenFlex*. In a subsequent study phase, 69% of these foods were reported by around 90% of the ethnic minority groups and contributed to 17% of their reported energy intake. However, differences between the three study groups in median self-reported energy intake remained (Sweden 7.19 MJ, Syria/Iraq 5.54 MJ, and Somalia 5.69 MJ). The groups also showed differences in relative energy contribution from fats and carbohydrates, as well as differences in energy intake from food groups such as bread and sweet snacks. We conclude that a dietary assessment instrument containing culture-specific foods could not resolve group differences in reported energy intake, although these foods provided content validity and contributed 17% of energy intake. The dietary habits collected in this study serve to develop new dietary assessment instruments.

## Introduction

The mnemonic Anthropometry, Biochemical markers, Clinical observations and Diet (ABCD) indicates the breadth in nutritional assessment and its associations with culture. Development of dietary assessment instruments for a culturally diverse population therefore requires attention with regards to choice of instrument (24-hour diet recall [24hDR], food frequency questionnaire [FFQ] or diet record), mode of administration (self-administration or interviewer-administered), range of included food items and portion size quantification.^(1,2)^ Studies need to take special care regarding recruitment strategies and language. Methods may furthermore depend on how long a population of interest has been in the country of residence. Such considerations relate to dietary acculturation, i.e. the adaptation process of an immigrant population to dietary habits of their host country and is related to a plethora of more or less modifiable reciprocal factors at the individual and societal level,^(3)^ such as psycho-social factors, environmental factors, and culture.

Existing national dietary survey data in Sweden, with 73% non-response from migrant groups (compared to 36% overall), hints at potential difficulties in assessing their eating habits.^(4)^ In this survey, reported energy intake and food group diversity among migrant groups are lower compared to participants born in Sweden (*unpublished results*).^(5)^ This could indicate that dishes and foods commonly consumed by ethnic minority groups are absent or not well-captured in the dietary assessment instruments, leading to biased estimates of intake. Consequently, associations between diet and health cannot be studied in those parts of the Swedish population where health inequalities are more likely, since dietary acculturation, as well as experienced nutrition transitions in country of birth, may have influenced the immigrant’s diet, and thereby their health.^(6–9)^

A variety of instruments have been used to capture diet among immigrant groups in Sweden. A study comparing elderly Iranians living in Sweden with Iranians living in Iran, describes differences in diet composition and food choices where immigrants reported less bread and more fruit, vegetables, dairy, and meat^(10)^; however, the FFQs were different in length (114 vs 168 items) and food groups had different emphasis. Diet among first-generation Iranian and Turkish women compared to Swedish women, reported a nearly 2 MJ/d lower energy intake assessed with four repeated 24hDRs.^(11)^ The diet of children from immigrant compared to non-immigrant parents showed small but significant differences^(12)^; however, the FFQ was not culturally adapted. Food records have been used to measure diet intake among Iraqi population with high risk of diabetes, but diet descriptions have focussed on nutrient intake and not food choices,^(13,14)^ thereby lacking the information needed for translation to public health. Misreporting of energy intake plays a role,^(11,13,15)^ but it is unclear how much of this is due to the instrument (lacking items or examples which are culturally adapted), unfamiliarity with participation in nutritional research or a true reflection of low intake when eating an energy-restricted diet.

Dietary assessment in a research population comprising a variety of ethnicities, preferably uses a dietary assessment instrument that is open-ended (e.g. diet records, 24hDR) as opposed to closed-ended questionnaires (e.g. FFQ, food screeners). Although online dietary assessment tools have provided great advantages in nutritional epidemiology,^(16)^ enabling low-cost dispersion of the instrument as well as fast analysis, they have as a disadvantage that they are strictly speaking no longer open-ended. A participant will have to find the food consumed in the list of foods provided by the instrument. To make best use of the advantages of the online ‘open-ended’ instruments, careful consideration of the food list in culturally diverse study groups is of importance.

This study aimed to develop and give content validity to new dietary assessment instruments and extend the content validity of an existing instrument (*RiksmatenFlex*, the 24hDR instrument developed by the Swedish Food Agency^(17,18)^) by involving ethnic minority groups in the food list adaptation. We describe the overall study design of “Mat i Sverige” (Eating in Sweden), the *RiksmatenFlex* adaptation, differences in instrument administration, and the consequences thereof for reported food and nutrient intake between participants born in Sweden, Syria or Iraq and Somalia.

## Methods

The study ran between December 2020 and January 2023 (Table 1). The COVID-19 pandemic affected recruitment, study protocol and study duration. This study obtained ethical approval from Swedish Ethical Review Authority, Gothenburg. Written informed consent was obtained from all participants.

**Table 1.**
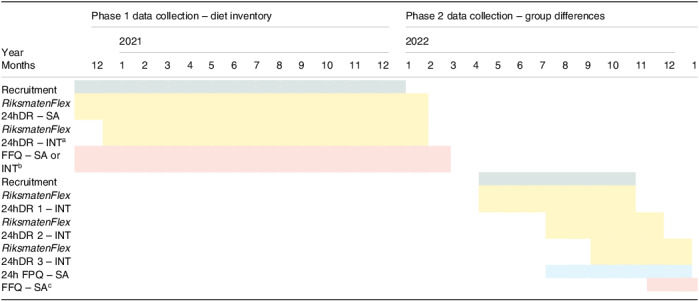
Study overview for “Eating in Sweden” (Mat i Sverige)

24hDR, 24-hour diet recall; FFQ, Food Frequency Questionnaire (semi-quantitative, i.e. including portion size quantification); 24h-FPQ, 24-hour Food Portion Questionnaire (1-day recall of a list with common foods, including portion size quantification); SA, self-administered; INT, interviewed.

a Only study groups born in Syria/Iraq and Somalia.

b SA for study group born in Sweden; INT for study groups born in Syria/Iraq and Somalia.

c Only study group born in Sweden.

### Participants and recruitment

With an anticipated start of a birth cohort requiring retrospective and prospective dietary assessment instruments administrable in a large sample size, the validation study included women over the age of 18 years who were pregnant or had at least one child born in Sweden and lived in the county of Örebro (Sweden). We aimed to recruit three equal sized study groups of women, originating from four countries: (1) Sweden (and whose parents were born in Sweden), (2) Syria or Iraq or (3) Somalia. The last two study groups were formed of three of the largest ethnic-minority groups receiving mother-child care in the county, 4.4%, 2.6%, and 3.6%, respectively, of all registered pregnancies.^(19)^ Participants were recruited in the community, such as family support centres (*familjecentral*), Swedish language classes, healthcare centres, schools, and community groups. For recruitment, we used posters, website, social media, personal contact, and local news media, focussing on living areas with a high proportion of immigration.

Due to COVID-19, routine indoor activities (e.g. play groups, information meetings) at the family centres were cancelled or relocated to outdoor activities which often had low(er) attendance, resulting in a long recruitment period. Communication problems because of language and cultural barriers made recruitment even more difficult. These difficulties were partly overcome by contacting potential participants through key people in the community or communicating with help of an (in)formal interpreter. Information about the study was available in Swedish, Arabic, and Somali. Participants could register themselves through a short online questionnaire which checked eligibility, obtained consent, and collected contact details. Where necessary, we assisted with this questionnaire.

National dietary survey data from 2010/11 included 10 women born in the Middle East.^(5)^ Their reported energy intake was 1.87 MJ/day (∼400 kcal) lower compared to the Swedish born sample. To be able to observe a 1.87 MJ/day higher energy intake, by extending the available food list with culture-specific food items, we needed to collect data on 40 women (90% power, 5% chance of type I error). Considering that participants could withdraw from the study at any time, we aimed to recruit 10% extra resulting in 44 participants in each study group (132 participants in total).

### Study design

Data collection was divided in two main phases (Table 1) and consisted of three main types of dietary assessment. In this publication, we focus on the 24hDR (*RiksmatenFlex*). We administered this instrument in two modes: self-administered and interviewed. Because of covid-19, the study protocol was changed to hold interviews online (zoom); however, when restrictions were eased, interviews were on request held at a public place.

During phase 1 of the study, all participants first self-administered their 24hDR (this also to test feasibility for the future birth cohort). Participants born outside Sweden were followed up with a video-interviewed 24hDR on the same day (i.e. we scheduled the interview date and time and asked participants to complete their self-administered 24hDR beforehand). The interviews included additional questions on food purchasing, preparation, and typical meal descriptions; the answers to which we used for instrument updates and development. Moreover, a preliminary FFQ was included (also interviewed, results not reported on here). The interviews were done by a single interviewer (SL) in Swedish. An interpreter was arranged on request. The total interview was scheduled to last about 2 hours, but when an interpreter was present, interviews took longer. Not all interviews could be completed within the scheduled time, but all included a complete interviewed 24hDR.

When phase 2 of data collection began, a second round of recruitment was started to compensate for study drop-out. Since data collection of self-administered 24hDR during phase 1 proved difficult, the study protocol was changed to three *interviewed* 24hDR instead, for *all three* study groups. Interviews were done by two interviewers in Swedish (ZA, ML) or Arabic (ZA). An interpreter was arranged on request. On completion of the study, participants received a food voucher for 200–300 kronor (about €20–30), depending on whether they took part in the interview in phase 1.

### Dietary assessment and variable derivation

Dietary data were collected using *RiksmatenFlex*, an online self-administered 24hDR designed and validated by the Swedish Food Agency for the national dietary surveys, only available in Swedish.^(17,18)^ During phase 1, this 24hDR was based on the version from the –at that time– latest completed dietary survey and consisted of 778 (partly composite) food items,^(17)^ linked to the Swedish Food Composition Database.^(20)^ Participants could find the foods and dishes by typing food or recipe names. Foods were recorded in 1-hour intervals. Portion sizes of reported foods could be quantified in household measures; alternatively, for amorphous foods (e.g. rice dishes), photos with up to six incremental portion sizes could be chosen. The interviewer searched for the best available alternatives and disaggregated dishes to account for culture-specific dishes not in the food list. The interviewed 24hDR were not recorded for video nor sound, but directly entered into *RiksmatenFlex* and notes were made on paper which were transferred to a database using a semi-structured data-entry form.

After phase 1, culture-specific foods and dishes reported by participants from Syria, Iraq, and Somalia were tallied and for 78 common foods and recipes a food composition was obtained. Recipes were based on the participants’ descriptions, aided with information from websites, food blogs, and cookbooks. A full list with added items and the context in which they may be eaten is available in S-Table 1.

During phase 2, we used *RiksmatenFlex* with the extended food list of 856 food items. We repeated the assessment three times over the course of a half year. The repeated measures enabled us to take day–to–day variation into account (as well as comparison against the FFQ with 6-month recall, not reported on here). Participants in all study groups were contacted by study staff (ZA, ML) to schedule their interview date and time in advance. We contacted participants via phone, mail, SMS or WhatsApp at different times of the day (including evening). Interviews were held online or at public places such as a family centre or library. We offered interview dates/times on all days of the week, between 08:00 and 20:00. Interviews were held in Arabic or Somali if requested. Interviews lasted a median of 27 minutes, with an interquartile range of 23–32 minutes.

The individual food items were grouped into 31 food groups (S-Table 1). The reported weights and energy contribution of the individual food items were summed by food group to represent the participant’s daily intake (phase 1) or by averaging the number of available 24hDR for an individual (phase 2).

Food group and nutrient intake data were checked for outliers in energy and macronutrient content. Outliers caused by portion sizes or an unusual low number of reported food groups or combinations of food groups were checked against interview notes for verification.

### Other variables

At recruitment we collected data on inclusion criteria, education (age until formal education was received), place of recruitment, and self-reported height and weight. During the interviews in phase 1, we additionally collected information on year of emigration from country of birth and year of immigration to Sweden. In phase 2, we modified the question regarding education and asked participants about their highest education obtained and repeated the self-reported anthropometry.

### Statistical analysis

Socio-demographic information from participants starting phase 1 and/or 2 are provided as median (interquartile range, IQR) for continuous variables or number (percent) for categorical variables.

**Fig. 1.**
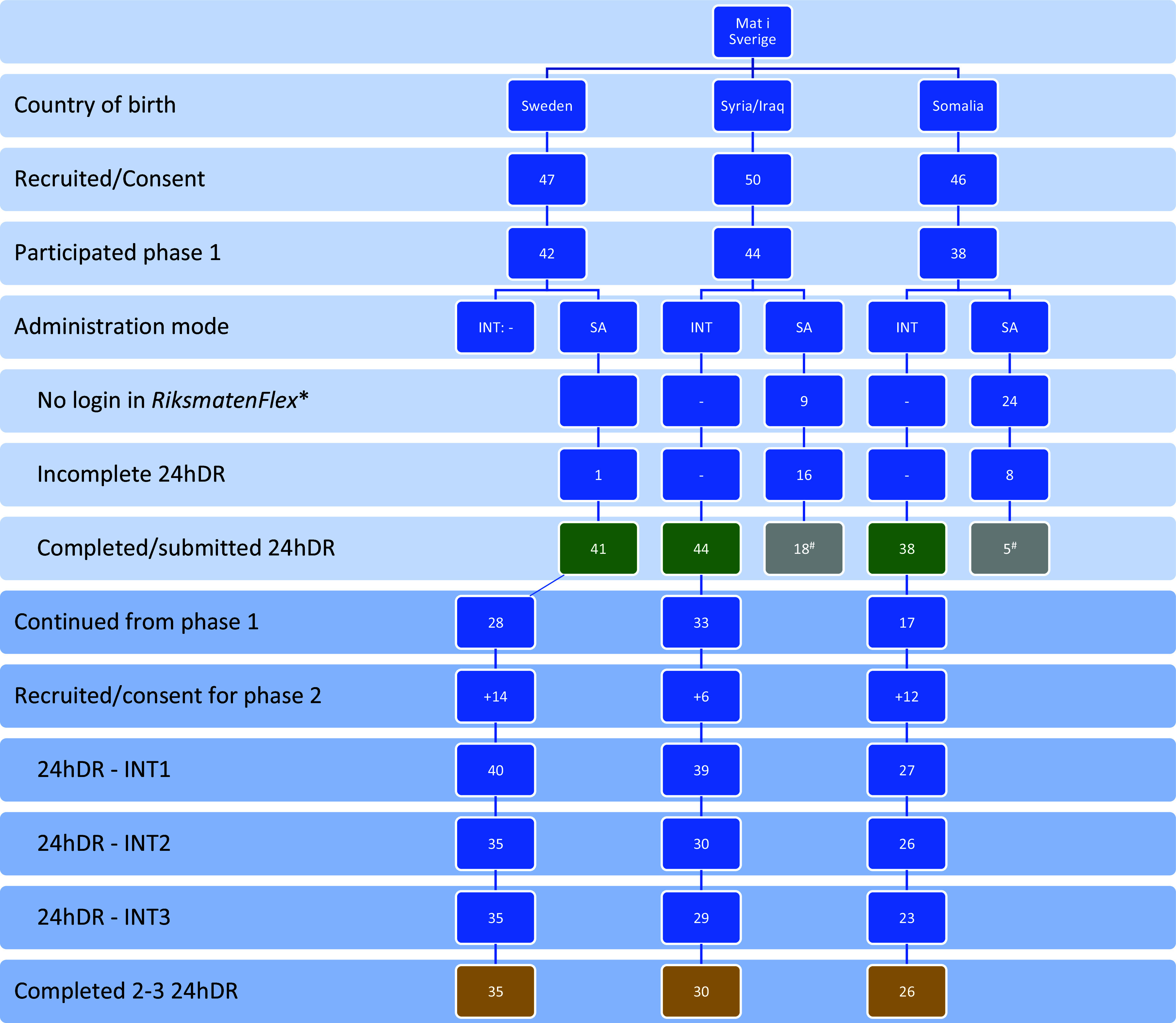
Flowchart of study participants included in the presented results. 24hDR, 24-hour diet recall; SA, self-administered; INT, interviewed. Green: Description of study group differences (N = 123). Grey: Comparison of self-administered versus interviewer-administered 24hDR (N = 23)^#^. Yellow: Repeated interviewer-administered 24hDR with the updated *RiksmatenFlex* food list (N = 91). Light blue rows: phase 1; darker blue rows: phase 2; indented lines indicate subcategories. *‘No login’ had different implications for the three study groups. For the group born in Sweden, it meant ‘no response’ and after two reminders, the participant was no longer approached (n = 5). No data apart from the recruitment information was available for this group and they were therefore *excluded* from the analysis. For the other two study groups, the interview was the determining factor for response. Those not completing the self-administered 24hDR but who participated in the interview were marked as ‘no login’; not completing either was considered ‘no response’ (n = 6 Syria/Iraq, n = 8 Somalia). ^#^2 participants (1 in each study group) submitted an empty 24hDR and were excluded from the analysis on administration comparison.

Where we compared differences due to administration mode (self-administered *vs* interviewed), median (IQR) of nutrient and food group intake are given (Fig. 1, grey). Agreement between administration modes on key nutrients (energy, macronutrients, and fibre) are depicted in Bland-Altman plots. Agreement between categorical variables was assessed using kappa-statistic.

For data collected during phase 1, differences between study groups regarding nutrient intake and food group consumption were tested using non-parametric median tests for continuous variables (followed by pairwise comparison if statistically significant) and Chi-squared test for categorical variables (Fig. 1, green).

For data collected during phase 2, only participants who participated in at least two (of three) interviewed 24hDR were included in the analysis (Fig. 1, yellow). For each study group, energy contribution of the foods added to *RiksmatenFlex* were quantified and median (IQR) of nutrient and food group intake described.

All analysis were carried out using IBM SPSS v28.

## Results

### Recruitment

Over the course of the study, we recruited a total of 175 participants, of which 143 during phase 1 (Fig. 1, S-Figure 1). When a participant discontinued or was no longer contactable, and the time left in the study phase allowed this, we continued recruiting to approach set targets of 44 participants in each group. At the start of phase 2, we recruited 32 additional participants to compensate for those lost. Over the whole study, most participants were recruited via family centres (19%), social media (18%), participant’s contact with friends, colleagues or family members (15%), Swedish language classes/adult education (12%), mother-child primary care (8%) or contact with study staff (8%). Family centres and language classes were especially good recruitment locations for the participants born in Somalia; social media for the participants born in Sweden; and contacts with friends, colleagues or family as well as language classes for participants born in Syria/Iraq.

### Phase 1: single self-administered and interviewer-administered 24hDR

#### Response

During phase 1 of the study (Fig. 1), we obtained consent from 47, 50 and 46 participants born in Sweden, Syria/Iraq and Somalia respectively, of which 42 (89%), 44 (88%) and 38 (83%) took part in phase 1. Participants had a median age of 34, 37, and 36 years and a median BMI of 23.7, 27.2, and 29.0 kg/m^2^ respectively (Table 2). Participants born in Syria/Iraq or Somalia had been in Sweden for a median of 9 and 12 years, respectively. More than two-thirds of these participants reported that their country of birth formed the basis of their food habits.

**Table 2.** Socio-demographic information of participants included in phase 1 (N = 124)

		Sweden					Syria/Iraq					Somalia				
		N	%	P25	P50	P75	N	%	P25	P50	P75	N	%	P25	P50	P75
Age (years)		42		30	34	38	44		32	37	43	38		31	36	40
Body weight (kg)		33		58	69	76	44		64	70	76	38		70	78	80
Height (cm)		34		163	165	170	44		158	160	164	38		157	163	166
BMI (kg/m^2^)		33		21.5	23.7	27.9	44		25.0	27.2	29.9	38		26.0	29.0	31.6
Time in Sweden (years)							44		6	9	18	38		8	12	18
Left country of birth	<1990						4	9				1	3			
	1990–2000						12	27				7	18			
	2000–2010						9	21				20	53			
	>2010						19	43				10	26			
Arrived in Sweden	<1990						2	5				0	0			
	1990–2000						4	9				6	16			
	2000–2010						13	30				18	47			
	>2010						25	57				14	37			
Interpreter present	No						30	68				31	82			
	Yes						14	32				7	18			
Country of birth formed basis of eating habits	No						11	25				6	17			
	Yes						30	68				25	71			
	Don’t know						3	7				4	11			

SA, self-administered; INT, interviewed, P, percentile.

Socio-demographic information for participants included in phase 2 can be found in S-Table 4.

The self-administered 24hDR was submitted by 41 (98%), 19 (43%), and 6 (16%) participants born in Sweden, Syria/Iraq, and Somalia, respectively, and an additional 1 (2%), 16 (36%), and 8 (21%) participants started but did not complete their self-administered 24hDR (Fig. 1). Of the 25 participants from Syria/Iraq and Somalia who submitted the 24hDR, 23 contained foods.

We completed 44 and 38 interviewed 24hDR for the participants born in Syria/Iraq and Somalia respectively. Evaluation of self-administered *RiksmatenFlex* during the interview revealed that the participants (multiple answers could be given): did not understand what was necessary (language related) or experienced the research process as difficult (45%), had not seen the reminders (22%), had no time/childcare (18%), or had problems with login procedure and/or program (13%). Five participants recalled their diet of the day of the interview rather than the day before the interview, resulting in incomplete data collection (one participant submitted their recall day and is included in the comparison study).

#### Description of study group differences (N = 123)

Since the response on the self-administered 24hDR was low, we describe nutrient and food group intake of the study groups using *different* administration modes of the *same RiksmatenFlex* version (Fig. 1, green).

The percentage of week versus weekend days were the same across the three study groups (Table 3). The median reported energy intake among the mothers born in Syria/Iraq and Somalia was lower compared to the mothers born in Sweden, though this did not reach statistical significance (P = 0.10). The median percentage of energy provided by protein was similar, whereas carbohydrate was highest (P = 0.004) and fat lowest (P = 0.022) among the Somali mothers compared to the other groups. Median fibre density was lowest in the Swedish group, but this did not reach statistical significance (P = 0.12).

**Table 3.** Nutrient and food group intake for participants with a single completed 24hDR in phase 1 (N = 123, combination of self-administered and interviewed)

	Sweden (SA, n = 41)					Syria/Iraq (INT, n = 44)					Somalia (INT, n = 38)				
	N	%	P25	P50	P75	N	%	P25	P50	P75	N	%	P25	P50	P75
Recalled day Mon–Thu	34	83				35	80				30	79			
Recalled day Fri–Sun	7	17				9	21				8	21			
Number of reported foods			13	16	23			14	18	23			16	20	25
Number of food groups			9	11	13			10	12	14			12	13	14
Reported energy intake (kJ)			4576	7222	9473			4252	5718	6920			4591	5673	7156
Reported energy intake (kcal)			1092	1725	2264			1016	1365	1654			1096	1356	1711
Fibre (g)			11.4	15.6	21.5			10.5	15.7	22.7			13.2	17.2	22.1
Alcohol (g)			0	0	0			0	0	0			0	0	0
Protein (en%)			13.7	17.3	19.8			14.8	17.4	21.8			13.8	17.3	18.8
Fat (en%)			29.8	34.1	41.0			30.8	36.6	39.3			23.4	31.9	34.2
Carbohydrates (en%)			40.2	44.5	48.9			36.4	43.9	51.2			42.2	50.0	58.4
Alcohol (en%)			0	0	0			0	0	0			0	0	0
Fibre (en%)			1.4	1.9	2.6			1.7	2.2	3.2			1.8	2.3	2.9
Beverages alcoholic^a^	3	7%	150	300	400	0	0%	.	.	.	0	0%	.	.	.
Beverages coffee	27	66%	400	500	800	29	66%	250	250	500	23	61%	150	250	500
Beverages sweet	16	39%	205	440	780	17	39%	157	207	315	19	50%	79	210	330
Beverages tea	13	32%	250	500	750	30	68%	250	250	400	27	71%	250	250	425
Beverages water	33	80%	400	600	800	41	93%	500	900	1200	37	97%	600	900	1350
Bread	29	71%	30	68	88	35	80%	40	98	142	28	74%	60	78	123
Breakfast cereals and porridge	19	46%	40	60	150	8	18%	23	45	100	14	37%	100	150	170
Cake, pies, biscuits, and desserts	17	41%	30	63	105	16	36%	12	42	84	9	24%	37	85	130
Cheese	22	54%	15	25	60	24	55%	9	16	25	22	58%	10	18	30
Dairy	33	80%	200	240	400	27	61%	52	160	283	32	84%	103	236	350
Egg	8	20%	82	104	164	22	50%	48	56	96	8	21%	56	76	104
Fish	13	32%	65	90	130	10	23%	130	175	300	11	29%	90	130	130
Fruit	24	59%	120	139	188	38	86%	123	145	245	31	82%	120	125	240
Legumes	3	7%	80	200	225	8	18%	22	52	100	7	18%	140	170	263
Meat	30	73%	55	137	225	32	73%	59	108	213	23	61%	90	100	200
Nuts and seeds	5	12%	17	18	90	12	27%	13	24	55	7	18%	10	10	17
Oil^b^	0	0%	.	.	.	9	20%	4	7	13	6	16%	13	13	20
Other	3	7%	2	4	6	6	14%	17	20	30	12	32%	16	30	30
Pasta, rice, and grains	30	73%	100	175	263	28	64%	105	163	245	28	74%	126	175	227
Pizza, pies, and takeaway	11	27%	95	170	220	2	5%	207	336	465	7	18%	240	300	310
Potatoes^b^	6	15%	113	135	140	5	11%	40	115	180	12	32%	43	58	120
Potato products and dishes	5	12%	111	168	252	2	5%	90	113	135	3	8%	45	50	65
Savoury sauces	16	39%	13	29	49	16	36%	21	33	70	15	39%	10	20	48
Snacks savoury	7	17%	14	15	36	5	11%	20	40	40	1	3%	4	4	4
Snacks sweet	16	39%	25	45	150	8	18%	10	26	75	3	8%	5	16	50
Soup	3	7%	150	225	400	7	16%	100	150	300	4	11%	75	163	263
Spreads and baking fat	19	46%	5	12	24	9	20%	4.5	6	6	25	66%	6	9	10
Sugar and sweet spreads	11	27%	11	20	34	15	34%	6	8	11	25	66%	8	10	27
Supplement	3	7%	12	24	100	1	2%	12	12	12	1	3%	19	19	19
Vegetables	21	51%	60	112	188	39	89%	85	179	304	33	87%	80	147	228
Vegetable products and dishes	4	10%	22	40	90	6	14%	75	250	375	5	13%	65	75	75

**Fig. 2.**
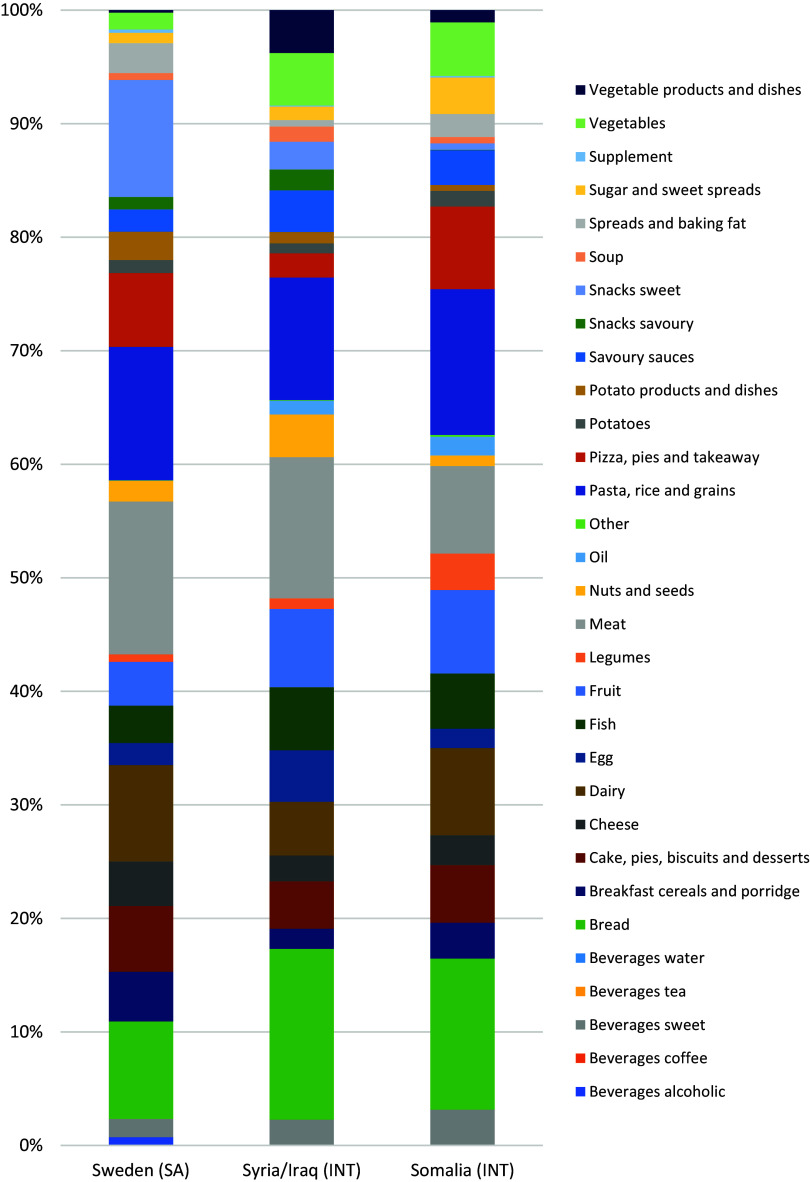
Contribution of food groups to mean daily energy intake (%) for participants with a single completed 24hDR in phase 1 (N = 123, combination of self-administered and interviewed). Food groups are sorted in alphabetical order (food group definitions in S-Table 1). SA, self-administered; INT, interviewed.

SA, self-administered; INT, interviewed; P, percentile.

a Food group intake is among consumers only (grams/day).

b See also notes in S-Table 1. A between group comparisons of the percent of food group consumers is shown in S-Figure 2.

The top five food groups which together contributed more than 50% towards energy intake among mothers born in Sweden were: meat, pasta/rice, sweet snacks, bread, and dairy (Fig. 2). Among mothers born in Syria/Iraq these were: bread, meat, pasta/rice, fruit, and fish. And for mothers born in Somalia: bread, pasta/rice, meat, dairy, and fruit. Foods such as vegetables and fruit contributed more towards energy intake among study groups born in Syria/Iraq and Somalia than Sweden. The largest differences in likelihood of reporting food groups were observed for: spreads, tea, sugar/sweet spreads, vegetables, and eggs (S-Figure 2).

#### Comparison of self-administered versus interviewer-administered 24hDR (N = 23)

**Fig. 3.**
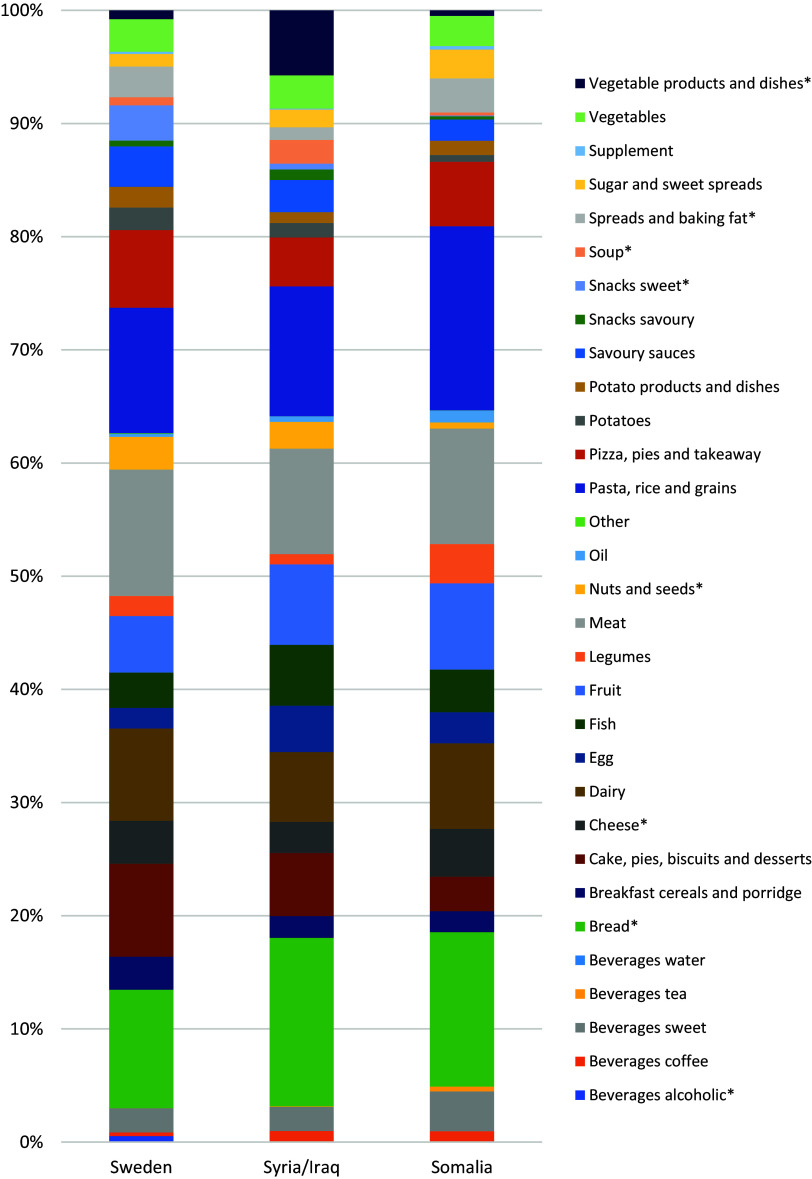
Contribution of food groups to mean daily energy intake (%) for participants with 2–3 completed 24hDR in phase 2 (N = 91, all interviewed). Food groups are sorted in alphabetical order (food group definitions in S-Table 1). Groups marked with * contributed with a significantly different median value to percent of daily energy intake).

Compared to the self-administered 24hDR (S-Table 2), the number of reported food items increased from 10 to 23 in the interviewer-administered 24hDR. Likewise, the median number of hours in which participants reported foods increased from 5 to 6 during the interview. In line with these observations, we observed an almost 1.5 MJ higher energy intake when the same participants were interviewed. Differences in nutrient intake are presented in Bland–Altman plots (S-Figure 3). These plots indicate a wide range on the Y-axis. Especially for fat and carbohydrates there was more disagreement with higher mean intake.

We compared the weights of reported food groups (among consumers only) against the results from the interviewed 24hDR (S-Table 3) and observed that the interviewed 24hDR recovered more occurrences in food group consumption for most food groups. For example, savoury sauces, sugar/sweet spreads, and cheese reported in the self-administered 24hDR were 22–36% of the reported occurrences in the interviewed 24hDR. Differences in reported food quantities between the administration methods (INT-SA) could be either positive or negative. For example, the median intake of cake/pies/biscuits/desserts in the self-administered 24hDR was 222% of the quantities compared to the interviewed 24hDR (but reported almost twice as often in the interviewed 24hDR); whereas median bread intake in the self-administered 24hDR was 86% of the quantities reported when interviewed (reported on by 18 participants in the interview compared to 9 during self-administration).

Before submitting their 24hDR, participants were asked to indicate whether the day was ‘usual’, ‘different from normal’ or whether they were ill. In both the self-administered and interviewed 24hDR, this was the participant’s evaluation of the day. None of the participants reported illness. A moderate agreement (kappa = 0.47) was observed in the participant’s experience of the day, where 4 changed from ‘usual’ to ‘different’ and 2 *vice versa*.

### Phase 2: Repeated interviewer-administered 24hDR with updated food list (N = 91)

Phase 2 of the study began with 40 (70% from phase 1), 39 (85% from phase 1) and 27 (63% from phase 1) participants born in Sweden, Syria/Iraq, and Somalia, respectively; of which 35 (88%), 30 (77%), and 26 (96%) completed 2–3 24hDR (Fig. 1, yellow). Socio-demographic information is shown in S-Table 4. Age and BMI distributions in phase 2 were similar to the distributions in phase 1. Large differences between the study groups were observed for education, where just over 75% obtained a degree in higher education for participants born in Sweden, whereas this was below 50% for the other two study groups. Participants born in Syria/Iraq and Somalia preferred more often to have face-to-face interviews.

In Table 4 the group differences in nutrient intake are presented. Median daily energy intake was statistically significantly higher among the group born in Sweden (P = 0.049). This group had also the highest energy contribution from fat and the lowest contribution from carbohydrates (P < 0.005). Median energy from protein intake was similar between groups as was fibre density.

**Table 4.** Nutrient intake for participants with 2–3 completed 24hDR in phase 2 (N = 91, all interviewed)

	Sweden (N = 35)			Syria/Iraq (N = 30)			Somalia (N = 26)		
	P25	P50	P75	P25	P50	P75	P25	P50	P75
Reported daily energy intake (kJ/d)	5930	7193	8509	4167	5542	7322	4676	5690	7352
Reported daily energy intake (kcal/d)	1417	1718	2034	996	1325	1750	1118	1360	1758
Fibre (g/d)	15.4	18.1	24.0	11.8	16.4	20.9	13.2	15.9	19.1
Alcohol (g/d)	0	0	0.2	0	0	0	0	0	0
Protein (en%)	14.0	16.7	19.1	15.6	16.8	18.2	15.4	16.9	18.8
Fat (en%)	34.7	37.8	41.3	30.7	33.4	36.9	27.0	29.5	33.6
Carbohydrates (en%)	39.6	42.3	46.4	42.9	48.0	50.3	45.6	50.8	54.6
Alcohol (en%)	0	0	0.1	0	0	0	0	0	0
Fibre (en%)	1.9	2.1	2.5	1.8	2.3	3.0	1.9	2.2	2.7
Energy intake from NEW foods (kJ/d)	0	0	10	466	905	1434	532	966	1558
Energy intake *not* from NEW foods (kJ/d)	5920	7193	8423	3010	4209	6300	3433	4993	5965
Energy intake from NEW foods (% of daily energy intake)	0	0	0.2	11.1	16.8	27.8	7.2	17.2	25.9
Reported foods (number/d)	17	21	25	16	17	20	16	19	21
Food groups (number/d)	11	12	13	10	11	12	10	12	13

P, percentile.

Of the 78 culture-specific foods added in phase 1 (S-Table 1), 54 (69%) were reported on in phase 2. The five most common foods were: Oriental thin bread, spiced coffee with sugar, suugo suqaar (a Somali meat stew), bariis (Somali rice dish), and kubbe/kibbeh (oriental bulgur parcels). In the group born in Sweden the added foods were reported by 9 (26%) participants, whereas this was 29 (97%) and 23 (88%) from the study groups born in Syria/Iraq and Somalia respectively. Among the latter two groups, culture-specific foods contributed maximally 75% to reported daily energy intake in a single 24hDR. Based on 2–3 24hDR, a median (IQR) of 0% (0–0.2%), 17% (11–28%), and 17% (7–26%) of daily energy intake was accounted for by the culture-specific foods (Table 4). There were no statistically significant differences in the contribution towards daily energy intake from the culture-specific foods between those recruited in phase 1 and phase 2 of the study (Syria/Iraq phase 1, n = 24: 18% (11–28%), phase 2 n = 6: 14% (12–28%); Somalia phase 1, n = 16: 17% (5–24%), phase 2 n = 10: 20% (7–32%)).

After re-grouping the culture-specific food items into their respective food groups, we studied the main energy contributors in the three study groups (Fig. 3). The top five energy contributors ranked nearly the same as in study phase 1 and were meat, pasta/rice, bread, cake/pies/biscuits/desserts, and dairy for mothers born in Sweden; bread, pasta/rice, meat, fruit, and dairy for mothers born in Syria/Iraq; and pasta/rice, bread, meat, fruit, and dairy for mothers born in Somalia. Statistically significant differences between study groups were observed in energy contribution from food groups such as bread (and related groups such as spreads and cheese), sweet snacks, nuts, and vegetable dishes. Borderline non-significant differences existed for pasta/rice and eggs. The food group frequency and weight are shown in Table 5.

**Table 5.**
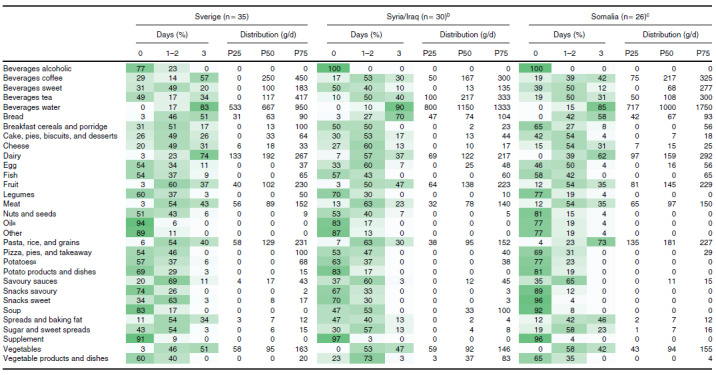
Frequency of reporting (%) and food group intake (grams) for participants with 2–3 completed 24hDR in phase 2 (N = 91, all interviewed)

P, percentile.

The distribution of food group weight is for the whole study group (for non-consumers and for those consuming the food group on 1, 2, or 3 days).

a A definition of the food groups is provided in S-Table 1, including context of eating which may explain differences in food groups marked with.

b 1 participant completed two 24hDR.

c 3 participants completed two 24hDR.

## Discussion

After a difficult recruitment process, this study identified 78 foods commonly reported by women born in Syria, Iraq, and Somalia who have lived in Sweden for approximately 10 years. In a subsequent study phase, nearly 70% of these foods were reported by around 90% of the study groups born in Syria/Iraq and Somalia contributing nearly a fifth towards reported daily energy intake, indicating content validity. However, our hypothesis that the addition of these foods would resolve the lower reported daily energy intake compared to the study group consisting of women born in Sweden, was not confirmed. The three study groups show differences in the relative contribution from fats and carbohydrates to daily energy intake, as well as differences in reported food groups such as bread and sweet snacks.

To stimulate participation in the study, we applied a range of strategies to lower language barriers and enhanced personal communication where possible. The study’s recruitment progress was nevertheless slow and affected by COVID-19. Challenges in the recruitment process have also been described by other Swedish studies aiming to be inclusive of multiple ethnicities.^(4,11,14)^ Although we were unable to reach recruitment targets, we reached out to participants in their familiar environments and succeeded to include those with limited Swedish language skills and lower educational attainment.

This study had several strengths and weaknesses. First, participants were recruited from community settings, not from outpatient or primary care facilities thereby lowering the risk that personalised dietary advice had been received and changes in usual dietary patterns had occurred. However, some advice regarding diet will have been provided by healthcare staff to all participants during their pregnancy care and attendance of visits for childcare, which is standard in Sweden. Second, we could not match ethnicity between interviewer and respondent for a substantial proportion of interviews. Matching is often assumed to improve validity during data collection, although much is still unclear.^(21)^ A study held in Mississippi which focussed on diet misreporting, observed no statistically significant associations between those matched versus unmatched for ethnicity.^(22)^ Thirdly, to keep within the scheduled interview time as much as possible, we only asked about crude descriptions of culture-specific dishes and relied on published recipes for estimation of nutrient content. This may have resulted in ‘more complicated’ or mixed culture versions of otherwise common dishes.^(1)^ Fourthly, we did not repeat the phase 1 version of *RiksmatenFlex* during phase 2. This may have obscured a learning effect on the side of the interviewers. Lastly, the scheduled study break in between phases 1 and 2 (Table 1), resulted in loss to follow-up. However, we recruited new participants for all three study groups and thereby also partly avoided auto-correlation. Moreover, we observed similar contributions to energy intake from the newly added culture-specific foods among those participating in both phases of the study and those only participating in phase 2 (thereby not involved in the identification of the foods to add to the *RiksmatenFlex* system).

Some of the food group differences observed during phase 1 (e.g. vegetables and potatoes, see Table 3) can be explained by differences in administration mode. The interviewer disaggregated recipes which were unavailable in *RiksmatenFlex*, whereas the self-administrating group was more likely to find composite dishes in the food list which would have been differently grouped. The same may also apply to the differences observed in oil consumption; however, oil was also reported as an alternative to spread and differences may therefore reflect cultural differences (S-Table 1).

During the first phase, we assessed diet among the ethnic minority groups twice, once self-administered and once interviewed, where 43% and 16% of the Syrian/Iraqi and Somali participants respectively could login and complete the 24hDR on their own. Support relating to login procedures and availability of appropriate food and portion size choice were insufficient. Especially searching for more generic food items when a specific food item could not be found in *RiksmatenFlex* was perceived difficult (e.g. participants used brand names or searched for chicken-based alternatives where pork-based is more common in Sweden). Similar issues have been reported by others using self-administered 24hDR.^(23,24)^ As intended, we show Bland–Altman plots for comparison of the two administration methods; however, the reasons for non-completion of the 24hDR are equally important results and relevant for our future large-scale data collection in a birth cohort where we will be unable to interview everyone and subsamples may need to be created to interview participants. We will in later publications compare other administered short- and long-term questionnaires (Table 1) against the repeated 24hDR using the adapted *RiksmatenFlex*. These instruments have more straightforward login, less typing of text answers but retain the option to free text answers, thereby structuring data collection, but keeping some flexibility in reporting. Having developed these structured instruments and the food list update in *RiksmatenFlex* in tandem has given comparable food items, providing information on recent as well as long-term retrospective exposures. Capturing these exposures combined has been shown by others to have methodological advantages.^(25)^

A recent study compared the Automated Self-Administered 24hDR (ASA24) with and without assistance and observed that this did not greatly affect accuracy among women with low socio-economic status.^(26)^ Contrary to this, our study indicated large differences in nutrient intakes and number of reported foods between administration modes in phase 1 (S-Table 2). This may be explained by participant selection, since we did not exclude those with low Swedish language skills or computer literacy. Using the extended food list in phase 2, the lower observed energy intake among the women born in Syria/Iraq and Somalia compared to Sweden, may be explained by reporting bias (social desirability), alternatively it could be a true estimate (adhering to an energy restricted diet). Social desirability bias may have played a larger role among the women born in Syria/Iraq and Somalia, since their interviews were more likely held in-person, rather than web-based. Interviewer-administration of 24hDR has been associated with misreporting due to social desirability.^(27)^ Also educational factors, not ethnicity, matters in the expression of social desirability.^(28)^ However, our results show associations in opposite direction from previously observed, since lower education was associated with lower energy reporting. Selective reporting could not be observed in an unobtrusive diet observation compared to an interviewer-administered 24hDR,^(24)^ whereas a self-administered *vs* interviewer-administered web-based 24hDR showed lower agreement for cake/biscuits and spreads compared to other food groups.^(23)^ We recovered a smaller portion size of cake/pies/biscuits/desserts during the interview rather than self-administration (S-Table 2); however, the number of occurrences for the foods was higher when participants were interviewed. When comparing obtained energy and macronutrient intake between our study with that from a study in Sweden also using 24hDR among Turkish and Iranian women,^(11)^ we note that in both studies energy intake was lower, percentage energy from carbohydrates higher and a higher BMI was observed in the ethnic minority groups. Reasons for this could be weight consciousness (i.e. aiming for weight loss) which may be an alternative explanation of our observations regarding lower energy intake. We did not use biomarkers or assessed physical activity, nor did we specifically ask about intended weight loss through diet (to avoid perceived ‘labelling’ and reporting bias). No information on pregnancy or breast-feeding status was recorded during phase 1 of the study and all anthropometry was self-reported, hampering application of predictive equations to estimate energy requirements. Indeed, 30–42% of the study population were pregnant or lactating (S-Table 4), but the range in these group prevalences appear unrelated to self-reported median energy intake (Table 4). For comparison of dietary habits between study groups, we used percent energy contribution from food groups to remove differences due to absolute differences in intake (although selective food group reporting could still have occurred). In contrast, interviewing did not appear to affect energy reporting among the ethnic Swedes. This study group had similar median reported energy intake during phase 2 (video interviewed, Table 4) compared to phase 1 (self-reported, Table 3); however, the groups were not entirely the same (70% of the participants overlapped) and more than a year had passed between assessments.

We have reported on the differences in food group intake between study groups. The aim of the study was not to establish a representative individual’s intake of nutrients and/or food groups. Moreover, sampling was not aimed in obtaining national representation. Nevertheless, providing a comparison of the included population groups using the same instruments, recruited from the general population, with inclusion of participants who required language help and the inclusion of food group results in addition to nutrients, does provide added value to the presented results. Future analysis may focus on disaggregation of dishes to capture food weight more accurately as well as including objective markers of dietary intake and nutritional status.

We studied participants in a limited timeframe after having lived a median of 10 years in Sweden. During the interviews we obtained insights in dietary acculturation through reporting on common dishes and meals, of which 69% were reported on in subsequent 24hDR. This indicated that we had added foods which were commonly consumed, giving content validity to the *RiksmatenFlex* food list. A review of dietary habits of Arabic-speaking immigrants and refugees indicates that both low and high acculturation are associated with more healthy food choices.^(6,7)^ This emphasizes the importance of inclusion of traditional foods for preventing systematic misclassification of dietary habits among those who adhere to a more traditional pattern.

We conclude that choice of administration mode is important in reaching intended study populations. Addition of culture-specific foods to the food list of a web-based 24hDR did not resolve lower energy reporting among ethnic minority groups, but these foods gave content validity and made up 17% of a participant’s reported daily energy intake and may give an insight in dietary acculturation.

## Supporting information

Lentjes et al. supplementary material Lentjes et al. supplementary material

## Abbreviations

**24hDR:** 24-hour diet recall; **SA:** self-administered; **INT:** interviewed; **FFQ:** food frequency questionnaire; **P:** percentile; **IQR: inter-quartile range.**

## Supplementary material

The supplementary material for this article can be found at https://doi.org/10.1017/jns.2024.72
