# Supplementary material for: Development of dietary assessment instruments which can take cultural diversity and dietary acculturation into account: eating in Sweden (‘Mat i Sverige’)
Source: J Nutr Sci. 2024 Nov 15;13:e70. doi: 10.1017/jns.2024.72 (PMC11588418; doi:10.1017/jns.2024.72)
Supplement: Lentjes et al. supplementary material [file S2048679024000727sup001.pdf]

Development of dietary assessment instruments which can take cultural diversity and dietary acculturation into account: Eating in Sweden ('Mat i Sverige')

Marleen AH Lentjes, Sarah Lönnström, Karin Lobenius Palmér, Zeinab Alsammarraie, Anna Karin Lindroos, Jessica Petrelius Sipinen, Afsaneh Koochek, Robert Jan Brummer and Scott Montgomery

Supplemental materials

Table 1: Description of the added culture-specific foods, other changes made to RiksmatenFlex and the food group classification. 2

Table 2: Nutrient intake from a single self-administered compared to a single interviewed 24hDR during phase 1 (N=23). 11

Table 3: Reported food groups from a single self-administered compared to a single interviewed 24hDR during phase 1 (N=23). 15

Table 4: Socio-demographic information of participants included in phase 2 of "Mat i Sverige" (N=91, completing 2-3 interviewed 24hDR using the updated RiksmatenFlex). 17

Figure 1: Recruitment and participation during "Mat i Sverige". 9

Figure 2: Radar diagram of percent food group reporters in the single 24hDR during phase 1 (N=123, combination of self-administered and interviewed). 10

Figure 3: Bland-Altman plots for energy providing nutrients comparing self-administration against interviewed 24hDR (phase 1, N=23). 12

Table 1: Description of the added culture-specific foods, other changes made to RiksmatenFlex and the food group classification.

Based on the interviewed 24hDR and additional questions where we asked participants to describe a typical breakfast, lunch, dinner and between meal snacks, we identified 78 foods and dishes to be added to the *RiksmatenFlex* dietary assessment instrument.

Non-composite food items which were readily available in the Swedish food composition database were added, which included sesame oil and sliced chicken sausage. Other food items were added as alternative names to existing foods because of lack of food composition data, such as: Arabic style cheeses (as white cheese), yerba/mate (as green tea), pomegranate syrup and date syrup (as syrup), freekeh/firik and kamedi/qamedi (as generic grain). For yet other foods we changed the range of portion sizes available, such as for fresh green herbs (e.g. parsley, which may be eaten in salad-like quantities). Data-entry boundaries were adjusted (*e.g.* number of dolmas or cheese cubes). Lastly, different presentation forms of food items were added (*e.g.* seeds including shell).

To estimate nutrient composition of the culture-specific foods, we searched for recipes in cookbooks and various websites based on the short descriptions provided by participants during their interview [1–3]. The Somali recipes included were among others: meat stews with beef or chicken, pancakes, breads, legume dishes and sweets. Eastern Mediterranean dishes included: breads, stuffed vegetables, rice dishes, oriental pizza and samosas.

For new recipes, portion sizes were linked to existing photographs [4], based on recipe calculation, available commercial products or similar recipes or food items already in the food list. Other amendments included: alternative food or recipe names and the addition of major recipe ingredients as search terms to increase the chance of finding a match with more traditional dishes (e.g. searching for ‘beans’ would result in a short list including green beans, Mexican dishes [a Swedish adoption of ethnic dishes [5]], but also the Somali ‘*cambuulo/ambuulo’* or the eastern Mediterranean dish ‘*fool/foul*’).

Items were combined into food groups based on the context in which the food item was likely to be used and not always on botanical likeness (e.g. apple sauce was grouped with jam since it is used in similar context, quinoa with pasta/rice/grain dishes since it is eaten as a carbohydrate replacement). Composite dishes were grouped by main ingredient (e.g. dishes including pasta/rice/grains were grouped as rice/grain dishes, even if they contained meat and/or fish).

Food group	Description	Food groups notes/exceptions	Culture-specific foods added and context of eating
Beverages, alcoholic	Alcoholic beverages (any vol%)	Includes alcohol free variants	
Beverages, coffee	Coffee, filter as well as instant	Excludes coffee served with large quantities of milk	Arabic/Somali coffee with spices and sugar
Beverages, sweet	Lemonade, fizzy drinks, juices of fruit and vegetables	Includes diet/light beverages	
Beverages, tea	Black, green and herbal teas		Somali tea (shaah) cooked with milk, spices and sugar<br>Maté/Yerba (added as search name to green tea)
Beverages, water	Tap and bottled water		
Bread	Soft and dried bread, all grain types, rolls and sliced bread		Chapati<br>Fried pita bread (used as topping on soup, or in salads, e.g. fattoush)<br>Sabayaad (Somali flat bread, pancake style)<br>Muufo (Somali bread bun with corn, fried)<br>Samoon/samoun (soft white Oriental bread)<br>Thin bread (Oriental bread used for wrapping food or pick-up mouthfuls of food from a plate)
Breakfast cereals and porridge	Sweetened and unsweetened variants, all grain types	Includes bran products	
Cake pies biscuits and desserts	Biscuits, cakes, sweet rolls/bun, cream filled cakes, ice cream	Includes ingredients which are likely part of desserts/cakes (e.g. sweetened cooked fruits, pastry)	Basbousa (Oriental manna-based cake with sugar syrup)<br>Bread filled with dates, sweet<br>Bur saliid (Somali fried dough balls)<br>Icun (Somali cookie)<br>Mamoul (Oriental cookie filled with dried fruits, often dates)<br>Mamonia (Oriental milk/manna pudding)

Food group	Description	Food groups notes/exceptions	Culture-specific foods added and context of eating
			Halawat el jibn (Oriental sweet, mannadough filled with cheese filling)<br>Sisin (Somali biscuit with sesame seeds)<br>Xalwo (Somali sweet, has similarities with Turkish lokum)
Cheese	Hard and soft cheese, cheese for cooking	Excludes cheese in dishes (e.g. pizza)	Kaymak is a spread made from boiled cream (has similarities with 'clotted cream'). Large range in fat content depending on brand and conservation method (canned *vs.* fresh)<br>Existing food 'White cheese/feta' was used to capture a variety of Arabic-style cheeses (Akkawi, Moushallal [braided/string cheese])
Dairy	Milk, yoghurt (incl. Greek/Turkish), fil, quark, cottage cheese, cappuccino/latte	Includes plant-based alternatives<br>Excludes cheese	Ayran (yoghurt whisked with water/herbs, served with meals)<br>Labneh (thickened yoghurt, can have consistency of cream cheese, can be served in little balls with herb/seed coating)
Eggs	Eggs, omelette, soufflé		Shakshuka (eggs in spiced tomato sauce), may be eaten as breakfast
Fish	Fish and other seafood, dishes including fish/seafood, salads with fish		
Fruit	Fruits/berries, fresh, dried or cooked		**Note**: Bananas may be consumed as a condiment to Somali-style meals
Legumes	Beans, lentils, vegetarian alternatives, cooked dishes with beans, salads with beans, houmous, quorn	Excludes green beans and peas (see vegetables)	Cambuulo (Somali dish, mixture of beans and corn/rice, served with oil/sugar or yogurt; can be eaten as an evening meal)<br>Falafel or Bajiye (Somali version)<br>Fasolia without meat (Oriental bean stew)<br>Foul/fool (Oriental broad bean dish, cooked with herbs/-spices, tomato; may be served as breakfast)

Food group	Description	Food groups notes/exceptions	Culture-specific foods added and context of eating
			Mujadara (Oriental mixture of lentils and bulgur/rice, topped with *e.g.* fried onions, butter)
Meat	Beef, lamb, chicken, pork, game, sausages, offal, meat sauces/stews		Chicken sausage as sandwich filling (called 'mortadella' by our participants)<br>Digaag qumbe (Somali chicken stew with coconut)<br>Fasolia with meat (Oriental bean stew)<br>Fatteh/Fattoush with meat/chicken (fried Oriental-style bread with salad/vegetables)<br>Kebab, home-cooked (minced meat with vegetables)<br>Molokhiya/Mulukhiyah with chicken (Somali vegetable stew)<br>Sabanegh (Oriental spinach sauce/stew with meat)<br>Suugo suqaar (Somali beef stew [may be minced beef], vegetables [paprika], tomatoes, spices)<br>Tapsi, oriental chicken and vegetable stew (aubergine, potatoes, tomatoes)<br>**Note**: offal (kidneys/liver) may be consumed for breakfast.
Nuts and seeds	Roasted and unroasted, with and without salt, nut/seed spreads		Chia seeds<br>Sunflower seeds with salt (with shells)<br>Sunflower seeds with salt (without shells)<br>Pumpkin flower seeds with salt (with shells)<br>Pumpkin flower seeds with salt (without shells)<br>Tahini (used as ingredient in sauces/spreads)
Oil	All oils, including coconut		Sesame oil (macsaro, may be added to cambuulo or canjeero)<br>Za'atar with olive oil (may be used as a bread topping/dip)
Other	Cooking ingredients (lemon juice, salt, vinegar, cinnamon *etc*)		Za'atar (blend of sesame seeds with sumac and dried herbs, salt)
Pasta, rice and grains	Cooked dishes and salads, pancakes	Dishes may contain meat	Biryani with meat (Oriental rice dish with vegetables, topped with fruits/nuts)

Food group	Description	Food groups notes/exceptions	Culture-specific foods added and context of eating
			Biryani without meat<br>Bulgur/burghul cooked with oil, tomato purée<br>Canjeero (sourdough style pancakes, may be served with oil, meat stews)<br>Itch (similarities with tabouleh, with larger proportion bulgur)<br>Makloubeh with meat (Oriental layered 'upside down' rice dish)<br>Makloubeh without meat<br>Malawax (Somali pancake, this version is similar to Northern-European pancakes, may be served with butter, sugar, jam)<br>Somali rice dish (indicated as 'Bariis' [which is rice] cooked with oil/spices, may contain other ingredients such as vegetables)<br>Soor (Somali dish based on majsmjöl, porridge consistency, may be served as starch replacement in meal, has similarities with polenta)
Pizza, pies and takeaways	Pizza, shop-bought sandwiches, individual-type pies ('pirog')	Dishes may contain meat	Börek with meat<br>Kubbe/Kubba/Kibbeh (bulgur parcels, stuffed with meat/-onion mixture)<br>Oriental pizza (meat-based topping, may be served with raw vegetables on top)<br>Samosa/sambusa with meat<br>Samosa/sambusa without meat
Potatoes	Boiled or mashed potatoes		**Note**: Potatoes may be used in meat stews (as a vegetable)
Potatoes products and dishes	Cooked dishes and salads, pommes frites		

Food group	Description	Food groups notes/exceptions	Culture-specific foods added and context of eating
Savoury sauces	Cooked sauces, mayonnaise, tomato-based sauces, condiment sauces		Basbaas (Somali chilli sauce)<br>Yoghurt sauce with tahini and lemon (Oriental)
Snacks savoury	Crisps, savoury biscuits, taco crisps		
Snacks sweet	Chocolate, liquorice, sweets		
Soup	Meat and vegetable-based soups		Lentil soup (Oriental soup with lentils, may be combined with rice, spinach or lemon; may be served with fried bread)
Spreads and baking fat	Butter, margarine, diet margarine		
Sugar and sweet spreads	Jam, marmalade, honey, sugar, syrup		Pomegranate syrup (Oriental) was added as a search name to syrup (used on salads and as a bread topping; may also be combined with tahini as a spread)<br>**Note**: Sugar may be added to legume dishes ('cambuulo').
Supplement	Meal replacement, protein powder, aloe vera juice		
Vegetables	Raw and cooked vegetables, root vegetables, green beans/peas, mushrooms	Excludes potatoes	Molokhiya/Mulukhiyah (Jute mallow leaves, bought dried, used in stews)
Vegetable products and dishes	Cooked dishes and vegetable-based salads with dressing		Bamya, okra stew<br>Dolma or mahshi, vegetables filled with mixture of rice and meat or just meat (e.g. stuffed paprika, onion, courgette, aubergine, potatoes) with tomato sauce<br>Dolma or mahshi without meat<br>Fatteh/Fattoush without meat<br>Mtabbal (oriental dish, often aubergine, puréed with tomato, paprika, parsley)<br>Spinach sauce/stew (Somali/Eritrean dish)<br>Tapsi without meat

General notes regarding the 24-hour recalls in phase 1:
Lunch was described as 'cooked meal', 'larger meal', 'real food'; whereas evening meal as 'lighter meal' or 'prepared meal' or 'simpler to cook'.

It could be difficult for participants to differentiate between:

- 'sauce', 'soup' and 'stew'
- 'sweet', 'cookie' or 'bread'
- 'lemonade' and 'juice'

Common reported cooking fats:

- Deep-frying: rapeseed oil or sunflower oil
- Frying (meat, rice, vegetables etc): rapeseed oil or olive oil
- Baking: butter or baking margarine

References

1. Khadija Mohamud (2021) *Khadija's kök (Khadija's kitchen)*. Bonnier Fakta.

2. Mourtada Z (2018) *Zeina's kitchen Recept från Mellanöstern (Recipes from the Middle-East)*. Stockholm: Bonnier Fakta.

3. Hassan H & Turshen J (2020) *In Bibi's kitchen The recipes and stories of grandmothers from the eight African countries that touch the Indian ocean*. New York: Ten Speed Press.

4. Swedish Food Agency (2009) Portionguiden med nyckel. https://www.livsmedelsverket.se/om-oss/publikationer/sok-publikationer/handbocker-och-verktyg/portionsguiden-nyckel (accessed July 2023).

5. Solér C & Plazas MC (2012) Integration of ethnic food into Swedish food rituals. The cultural fitness of tacos. *Appetite* **58**, 928–935.

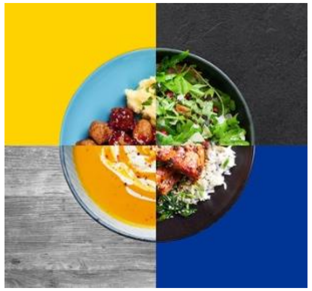

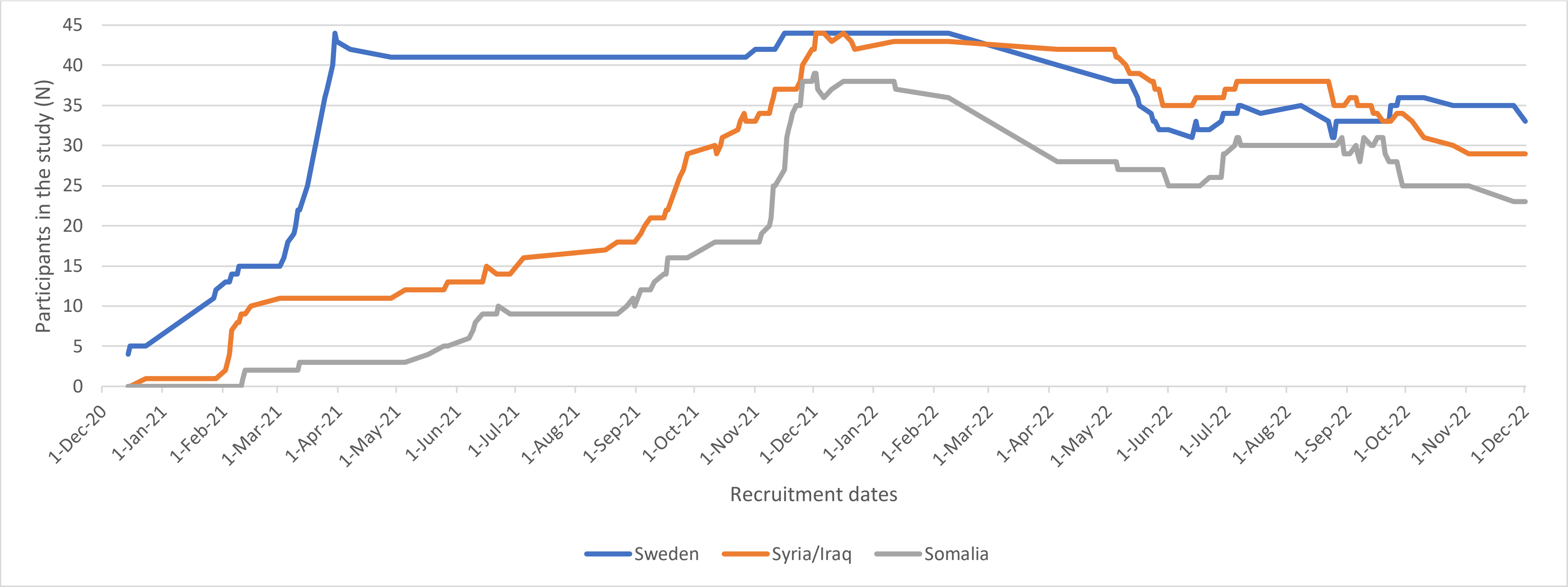

Figure 1: Recruitment and participation during "Mat i Sverige".

See also **Table 1** for study overview. Participants with whom we lost contact during the study had a study discontinuation date imputed (date which came closest to the announcements of the next study phase). E.g. a participant who completed phase 1, but did not do any of the three interviews of phase 2 was given a study discontinuation date in April 2022 when we started phase 2.

The sharp rise in recruitment April 2021 was due to a combination of factors: availability of a facebook page, local television, articles in two local newspapers (as well as our own recruitment activities in family centres etc).

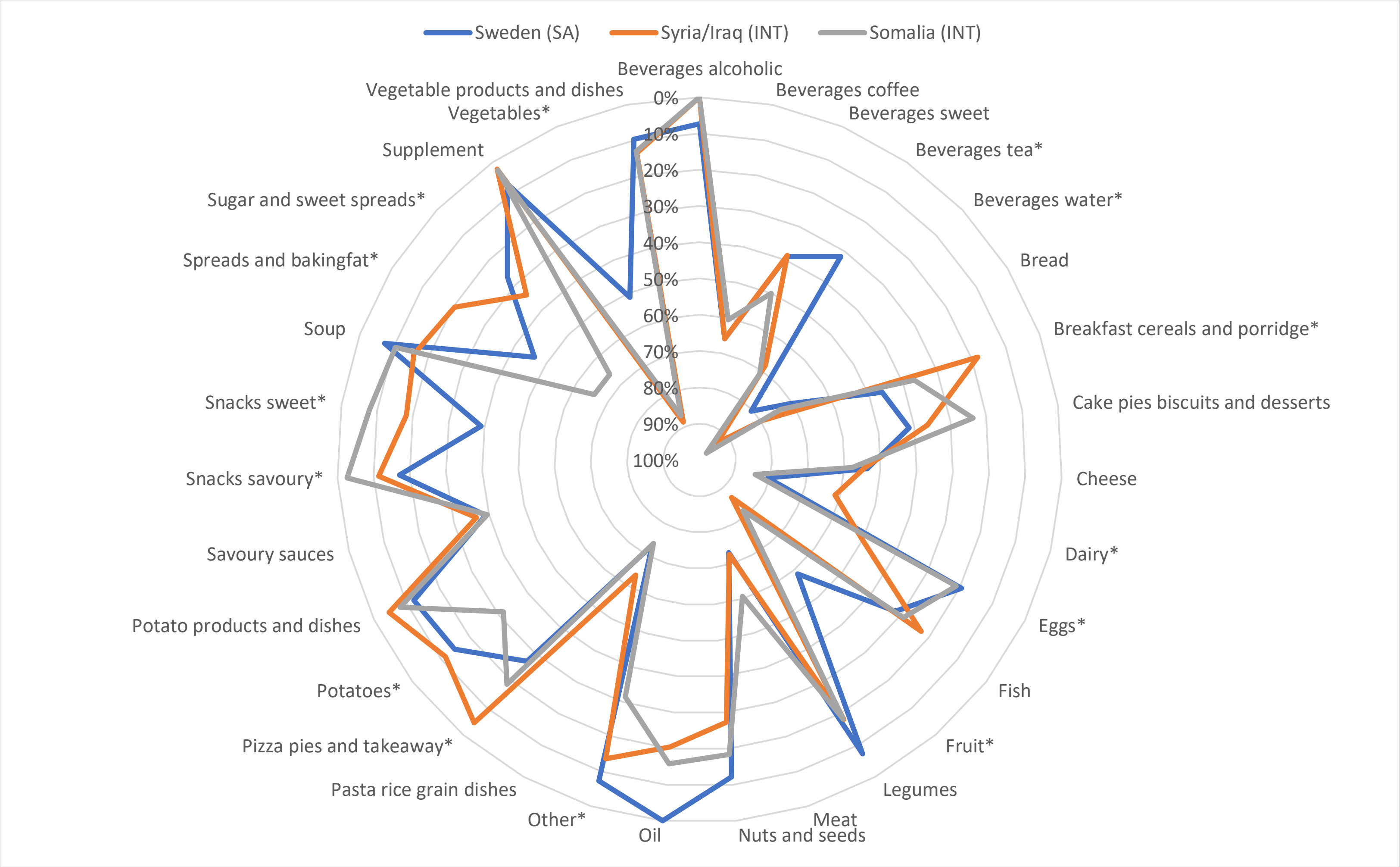

Figure 2: Radar diagram of percent food group reporters in the single 24hDR during phase 1 (N=123, combination of self-administered and interviewed).

24hDR, 24-hour diet recall; SA, self-administered; INT, interviewed. * Chi-squared test (P<0.05).
Food groups in alphabetical order. Note that the axis (0-100%) is in reverse order. Food group weights in **Table 3**.

Table 2: Nutrient intake from a single self-administered compared to a single interviewed 24hDR during phase 1 (N=23).

	**Self-administered 24hDR**				**Interviewed 24hDR**		
	**P25**	**P50**	**P75**		**P25**	**P50**	**P75**
Reported energy intake (kJ)	2462	4895	7847		4835	6337	8019
Reported energy intake (kcal)	588	1170	1875		1157	1514	1919
Protein (gram)	32	58	79		45	57	79
Fat (gram)	16	45	77		39	63	80
Carbohydrates (gram)	58	119	207		121	175	234
Alcohol (gram)	0	0	0		0	0	0
Fibre (gram)	5	10	19		14	22	24
Water (gram)	970	1573	2427		1449	2470	2731
Protein (en%)	15.7	19.7	25.1		14.3	17.0	18.8
Fat (en%)	20.2	36.6	40.7		31.2	36.4	38.9
Carbohydrates (en%)	38.6	45.4	52.0		38.6	46.1	49.7
Alcohol (en%)	0.0	0.0	0.0		0.0	0.0	0.0
Fibre (en%)	0.8	1.5	2.5		1.6	2.4	3.0
Fibre density (g/MJ)	1.0	1.9	3.1		2.0	3.0	3.7
Number of reported foods	6	10	15		15	23	25
Number of food groups	4	8	10		10	12	15
Number of reported hours with food/drink	3	5	6		5	6	7

24hDR, 24-hour diet recall; P, percentile.

Bland-Altman plots for energy providing nutrients are in **Supplement Figure 3.**

Figure 3: Bland-Altman plots for energy providing nutrients comparing self-administration against interviewed 24hDR (phase 1, N=23).

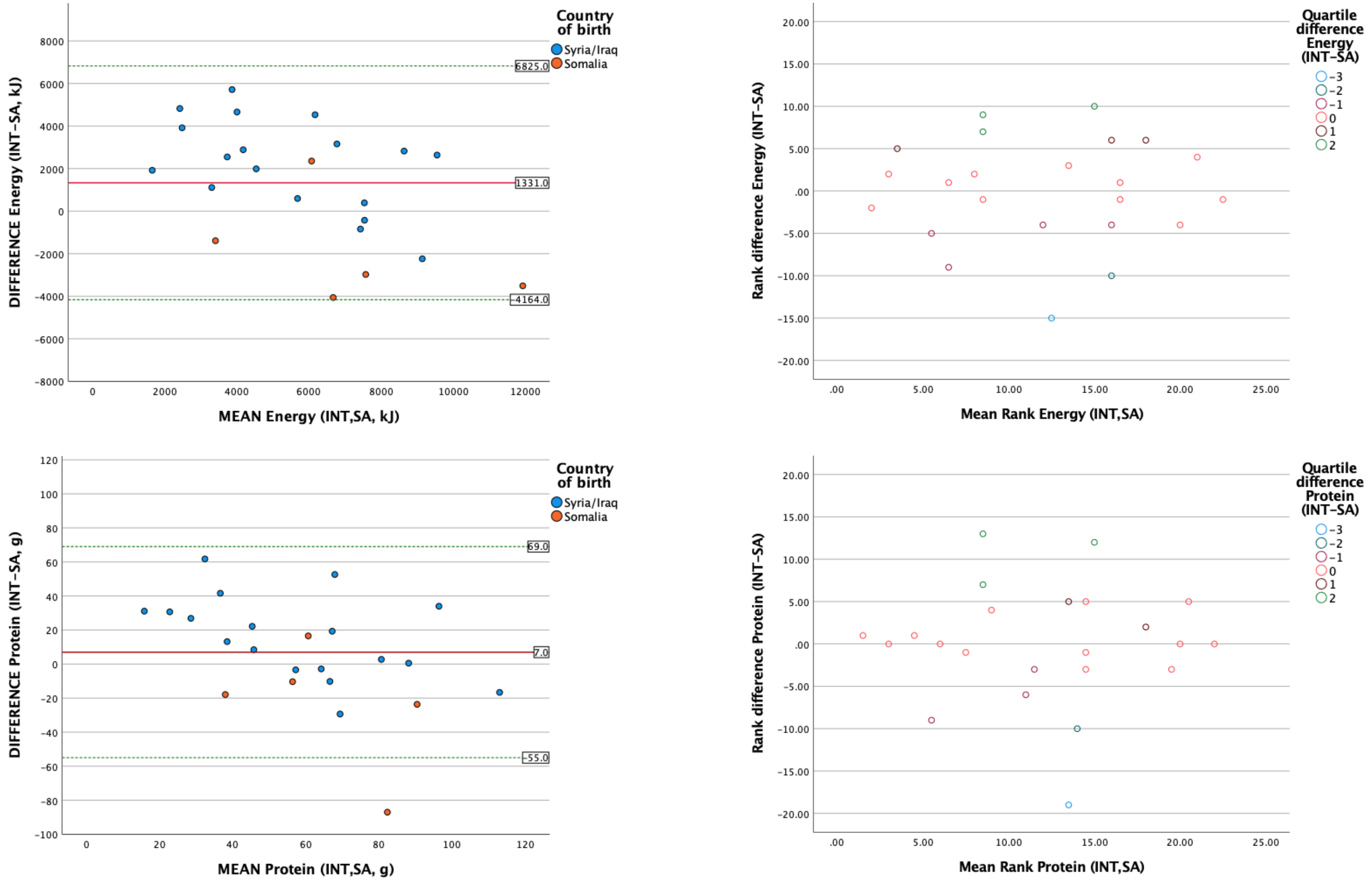

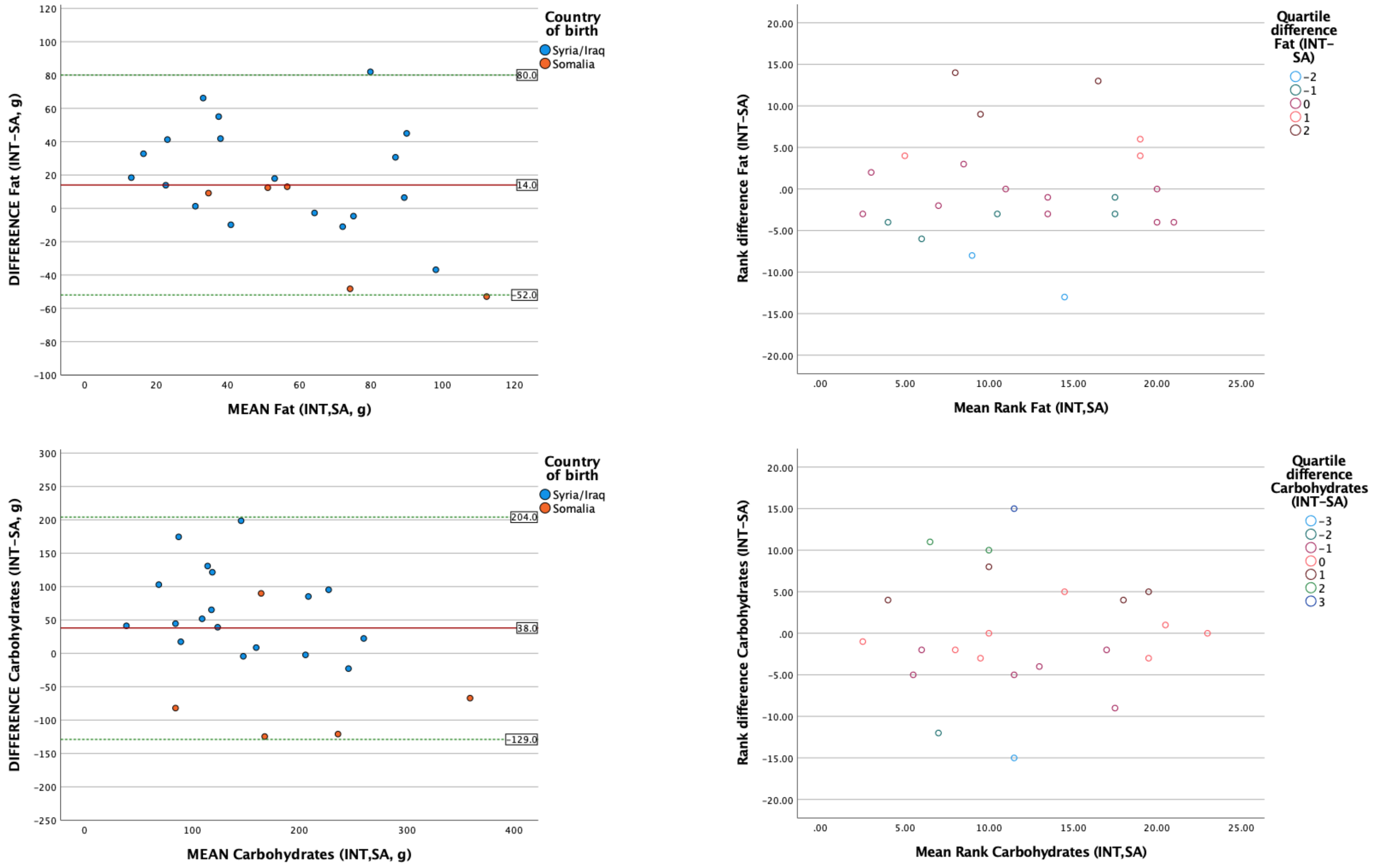

DIFFERENCE Fat (INT-SA, g)
MEAN Fat (INT,SA, g)
Country of birth
Syria/Iraq
Somalia
80.0
14.0
-52.0
Rank difference Fat (INT-SA)
Mean Rank Fat (INT,SA)
Quartile difference Fat (INT-SA)
-2
-1
0
1
2
DIFFERENCE Carbohydrates (INT-SA, g)
MEAN Carbohydrates (INT,SA, g)
Country of birth
Syria/Iraq
Somalia
204.0
38.0
-129.0
Rank difference Carbohydrates (INT-SA)
Mean Rank Carbohydrates (INT,SA)
Quartile difference Carbohydrates (INT-SA)
-3
-2
-1
0
1
2
3

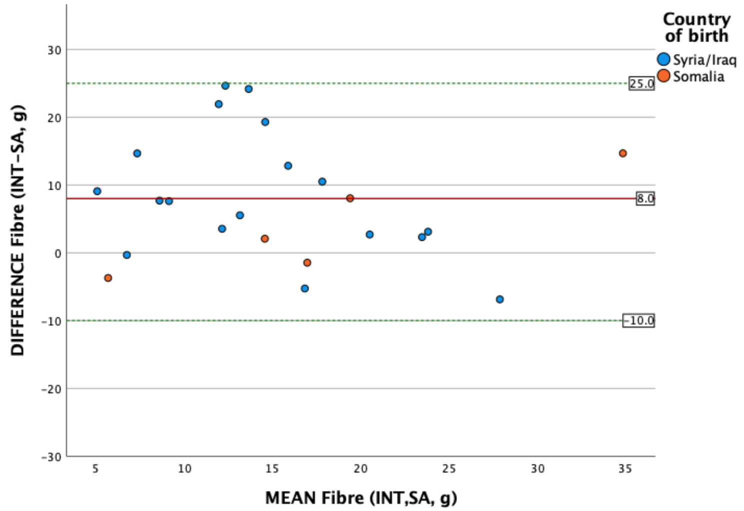

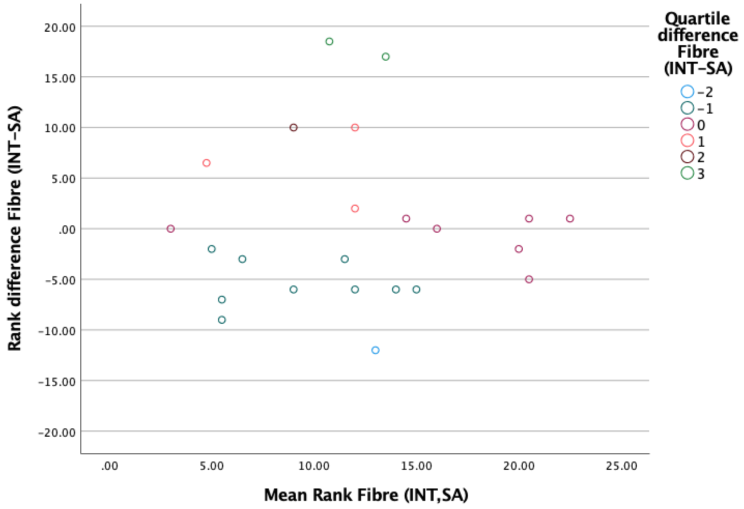

24hDR, 24-hour diet recall; SA, self-administered; INT, interviewed.

We studied agreement in reported nutrient intake of the self-administered (SA) compared to interviewed (INT) 24hDR using Bland-Altman plots. The red line is the mean bias (INT-SA) the green hashed lines are the reference interval (mean difference +/- 1.96*standard deviation of the difference). These plots show a wide range in reported intake on the Y-axis (INT-SA). Moreover, the scatter plots are funnel shaped or have a downward trend.

Analyses were repeated with the rank (1-23) of intake data obtained. A positive rank difference means that the participant ranked higher during the interviewed 24hDR than the self-administered 24hDR. The shift in quartile classification is indicated on a colour scale. Participants whose mean rank was either low or high were more likely to be in the same or adjacent quartile.

Median and interquartile range of energy and macronutrient intake for the self-administered and interviewed 24hDR are in **Supplement Table 2**.

Table 3: Reported food groups from a single self-administered compared to a single interviewed 24hDR during phase 1 (N=23).

	**Self-administered 24hDR**				**Interviewed 24hDR**				**Difference (INT-SA)**	
	**reporter (N)**	**P25**	**P50**	**P75**	**reporter (N)**	**P25**	**P50**	**P75**	**N [#]**	**Mean (g)**
Beverages alcoholic	0	.	.	.	0	.	.	.		
Beverages coffee	6	290	550	1050	13	250	300	500	6	-257
Beverages sweet	9	100	210	450	10	157	204	300	9	-43
Beverages tea	11	200	250	600	13	250	300	500	11	-5
Beverages water	16	375	500	1200	23	400	700	1200	16	150
Bread	9	75	88	120	18	40	102	150	8	35
Breakfast cereals and porridge	5	150	150	150	9	60	100	150	4	-42
Cake pies biscuits and desserts	4	89	144	294	7	10	65	130	4	-90
Cheese	4	19	22	26	11	8	20	33	4	-9
Dairy	14	100	200	384	16	134	225	288	11	-19
Egg s	9	48	96	96	12	38	96	112	9	9
Fish	3	7	300	335	2	90	188	285	2	-130
Fruit	13	85	120	250	20	120	158	263	13	12
Legumes	2	150	225	299	5	53	106	150	0	.
Meat	11	89	90	225	15	56	88	200	10	-16
Nuts and seeds	4	9	14	39	9	10	16	50	4	16
Oil	0	.	.	.	5	7	13	13	0	.
Other	0	.	.	.	6	15	17	30	0	.
Pasta, rice and grain dishes	11	105	308	472	15	105	175	315	10	26
Pizza pies and takeaway	6	160	235	300	2	310	388	465	2	-41
Potatoes	0	.	.	.	3	20	70	115	0	.
Potatoes, products and dishes	1	90	90	90	1	90	90	90	1	0
Savoury sauces	2	5	15	25	9	18	30	70	2	5

	Self-administered 24hDR				Interviewed 24hDR				Difference (INT-SA)	
	reporter (N)	P25	P50	P75	reporter (N)	P25	P50	P75	N #	Mean (g)
Snacks savoury	0	.	.	.	1	40	40	40	0	.
Snacks sweet	5	13	22	28	5	25	28	50	3	-49
Soup	3	150	375	450	3	100	225	300	2	-150
Spreads and baking fat	5	7	10	14	9	5	6	10	4	-19
Sugar and sweet spreads	2	6	23	40	8	9	13	36	2	-5
Supplement	1	19	19	19	2	12	15	19	1	0
Vegetables	8	99	172	200	21	75	170	312	8	93
Vegetable products and dishes	4	86	195	325	3	75	250	263	1	0

SA, self-administered; INT, interviewed.

* Reported food weights are for those participants who reported consuming a food item from the food group.

# Number over which the mean difference was calculated. This number may be different from the minimum value of the number of reporters in either administration mode, since the number of participants reporting on a food group may have been the same, but could still have been reported by different people in each administration mode (e.g. bread, soup, legumes).

A definition of the food groups can be found in **Supplement Table 1**.

Table 4: Socio-demographic information of participants included in phase 2 of "Mat i Sverige" (N=91, completing 2-3 interviewed 24hDR using the updated RiksmatenFlex).

		Sweden					Syria/Iraq					Somalia				
		**N**	**%**	**P25**	**P50**	**P75**	**N**	**%**	**P25**	**P50**	**P75**	**N**	**%**	**P25**	**P50**	**P75**
At study entry																
Age (years)		35		32	34	38	30		30	35	39	26		29	33	38
Body weight (kg)		29		61	68	86	30		63	69	75	24		68	79	80
Height (cm)		29		163	165	172	30		158	160	165	24		158	163	167
BMI (kg/m^2)		29		21.7	23.5	29.7	30		24.6	26.4	28.6	24		25.8	28.1	32.2
At end of study																
Body weight (kg)		35		60	73	85	29		64	70	77	23		65	75	88
Height (cm)		35		163	166	172	29		159	162	165	23		156	162	165
BMI (kg/m^2)		35		22.3	24.2	29.3	29		25.2	26.6	28.9	23		25.1	29.0	33.1
Pregnancy	Reported on in at least one interview	5	14%				3	10%				0	0%			
Breast feeding	Reported on in at least one interview	9	26%				6	20%				11	42%			
Digital interview	Interview 1	31	89%				21	70%				9	35%			
	Interview 2	31	89%				22	73%				12	#50%			
	Interview 3	33	94%				21	*72%				10	$44%			
Education	Don't know	0	0%				1	3%				0	0%			
	Did not go to school	0	0%				0	0%				0	0%			
	Swedish language classes	0	0%				3	10%				8	35%			
	Primary education	1	3%				1	3%				2	9%			
	Secondary education	5	15%				3	10%				6	26%			
	Applied (yrkeshögskola)	1	3%				3	10%				0	0%			

		Sweden					Syria/Iraq					Somalia				
		**N**	**%**	**P25**	**P50**	**P75**	**N**	**%**	**P25**	**P50**	**P75**	**N**	**%**	**P25**	**P50**	**P75**
	Komvux/Adult education	1	3%				5	17%				5	22%			
	Professional education, university	26	77%				14	47%				2	9%			
	Does not want to answer	0	0%				0	0%				0	0%			
Time in Sweden (years)							30		6	7	14	24		6	12	16
Left country of birth	<1990						1	3%				0	0%			
	1990-2000						8	27%				5	21%			
	2000-2010						4	13%				11	46%			
	>2010						17	57%				8	33%			
Arrived in Sweden	<1990						0	0%				0	0%			
	1990-2000						3	10%				3	13%			
	2000-2010						6	20%				10	42%			
	>2010						21	70%				11	46%			

* Missing values: n=1.

# Missing values: n=2.

$ Missing values: n=3.
